# Nutraceutical values of natural honey and its contribution to human health and wealth

**DOI:** 10.1186/1743-7075-9-61

**Published:** 2012-06-20

**Authors:** Abdulwahid Ajibola, Joseph P Chamunorwa, Kennedy H Erlwanger

**Affiliations:** 1 Department of Physiology, Faculty of Basic Medical Sciences, Olabisi Onabanjo University, Ikenne campus 121002, Ogun State, Nigeria; 2 Department of Veterinary Anatomy and Physiology, Faculty of Veterinary Science, University of Pretoria, Onderstepoort, South Africa; 3 School of Physiology, Faculty of Health Sciences, University of the Witwatersrand, 7 York Road, Parktown 2193, South Africa; 4 Department of Physiology, Faculty of Basic Medical Sciences, Olabisi Onabanjo University, Ikenne campus 121002, Ogun State, Nigeria

**Keywords:** Apiculture, Economic importance, Ethno-entomologist, Folk medicine, Health indices, Honey, Metabolic syndrome, Nutraceutics, Stingless bee

## Abstract

The use of natural honey (NH) as a nutraceutical agent is associated with nutritional benefits and therapeutic promises. NH is widely accepted as food and medicine by all generations, traditions and civilizations, both ancient and modern. The nutritional profiles, including its use in infant and children feeding reported in different literatures as well as health indices and biomarkers observed by various researchers are illustrated in this manuscript. The review documents folk medicine, experimentation with animal models, and orthodox medical practices shown by clinical trials. This covers virtually all human organs and body systems extensively studied by different workers. The sources and adverse effects of NH contamination, as well as the preventive methods are identified. This could promote the availability of residue free honey and a wholesome natural product for domestic consumption and international market. This could also help to prevent health problems associated with NH poisoning. In addition, apicultural practices and the economic importance of honey are well documented. This report also includes information about a relatively unknown and uncommon South American stingless bee species. We concluded this review by identifying important roles for Ethno-entomologists, other Scientists and Apiculturists in the development of stingless bees to boost honey production, consumption and economic earnings.

## Introduction

The use of natural honey as food and medicine by mankind has been in existence from time immemorial. In fact, records have it that raw honey is the most ancient sweetener, and it was noted to have been in use throughout the world several million years ago [1]. Natural honey (NH) is a sweet, flavourful liquid food of high nutritional value [2,3], and immense health benefits [3,4]. NH is produced by honey-bees as blossom honey by secreting nectars of flowers, and honeydew honey (forest honey) by secreting the exudates of plant sucking insects (Aphids). NH is widely embraced by all ages, and its use transcends the barriers of culture and ethnicity. The use of honey is even advocated and embraced by all religious and cultural beliefs.

NH is a liquid spoken of by all religious books, and accepted by all generations, traditions and civilizations, both ancient and modern. The religion of Islam recommended the use of honey as food and medicine, and even named an entire chapter in the Holy Qur'an called Surah al-Nahl meaning chapter of the Honey Bee [5]. In the book of hadith, Prophet Muhammad strongly advocated the use of honey for curative and healing purposes [6]. Furthermore, the Holy Qur'an explicitly encourages the consumption of NH as a highly nutritious and health promoting food in chapter 16 thus: “And your Lord inspired the bee(s), saying: "Take your habitations in the mountains and in the trees and in what they erect. Then, eat of all fruits, and follow the ways of your Lord made easy (for you)." There comes forth from their bellies, a drink of varying colour wherein is healing for men. Verily, in this is indeed a sign for people who think” [7]. In Christendom, there are references made to the importance of bees and honey in the Bible, and these include the Books of Exodus, Judges, Mathew and Proverbs [8-11]. In accordance with this Christian holy book, the Bible, King Solomon was quoted thus: “Eat honey my son, because it is good” [11]. In fact, it was reported in the Bible that John the Baptist actually thrived on a diet including wild honey for a long period of time when he was in the desert area or while travelling in the wilderness [10]. The other sets of people, sects, traditions and civilizations that attested to the popularity of honey include Budhists, Jews, Hindus and Vedas [1,12].

The profile of honey in the health shopping list is rising. The reason for this increased demand for NH is attributable to its popularity due to several medicinal uses that this substance has enjoyed throughout the history of mankind. It has been observed from time immemorial that NH is not only important for its medicinal attributes, but also useful as food sweetener, complete food and natural beauty agent [1]. NH has been used severally by the Tradomedical practitioners for numerous health benefits, and researchers have also documented honey’s beneficial roles in modern medicine, especially in wound treatments [13,14]. Several different surveys have been compiled on the nutritional and health aspects of honey [14-18]. However, the nutritional value and medicinal properties of NH are too numerous and highly inexhaustible to be comprehensively documented by these manuscripts. The records of honey as functional health food and uses of other honey-bee products are still incipient. Thus, the need to review some relevant materials on natural honey becomes imperative. This review documents the NH values as food and medicine, as well as enumerates the economic importance of NH production and other apicultural practices.

## Honey as food

### Nutritional profile

The composition of honey is mainly sugars and water (Table 1). In addition, it also contains several vitamins and minerals, including B vitamins as shown in Table 2. The other constituents of honey are amino acids, antibiotic-rich inhibine, proteins, phenol antioxidants, and micronutrients [2]. The sugars in honey are sweeter and give more energy than artificial sweeteners [2-4], and the most abundant sugar in honey is fructose (Table 1).

**Table 1:** Nutritional composition of honey*

	**Blossom honey**		**Honeydew honey**	
	**Range**	**Mean**	**Range**	**Mean**
Water	15 – 20	17.2	15 – 20	16.3
Total sugars		79.7		80.5
* Monosaccharides*				
fructose	30 – 45	38.2	28 – 40	31.8
glucose	24 – 40	31.3	19 – 32	26.1
* Disaccharides*				
sucrose	0.1 – 4.8	0.7	0.1 – 4.7	0.5
others	2.0 – 8.0	5.0	1.0 – 6.0	4.0
* Trisaccharides*				
oligosaccharides		3.1		10.1
erlose	0.5 – 6.0	0.8	0.1 – 6.0	0.1
melezitose		< 0.1	0.3 – 22	4.0
others	0.5 – 1.0	0.5	0.1 – 6.0	3.0
Minerals	0.1 – 0.5	0.2	0.6 – 2.0	0.9
Amino acids, proteins	0.2 – 0.4	0.3	0.4 – 0.7	0.6
Acids	0.2 – 0.8	0.5	0.8 – 1.5	1.1
pH value	3.2 – 4.5	3.9	4.5 – 6.5	5.2

*Data in g/100 g of honey, Adapted from [2,3].

**Table 2:** Chemical elements found in honey*

**Minerals**	**Amount (mg/100 g)**	**Vitamins**	**Amount (mg/100 g)**
Sodium (Na)	1.6 – 17	Thiamine (B_1_)	0.00 – 0.01
Calcium (Ca)	3 – 31	Riboflavin (B_2_)	0.01 – 0.02
Potassium (K)	40 – 3500	Niacin (B_3_)	0.10 – 0.20
Magnesium (Mg)	0.7 – 13	Pantothenic acid (B_5_)	0.02 – 0.11
Phosphorus (P)	2 – 15	Pyridoxine (B_6_)	0.01 – 0.32
Selenium (Se)	0.002 – 0.01	Folic acid (B_9_)	0.002 – 0.01
Copper (Cu)^a^	0.02 – 0.6	Ascorbic acid (C)	2.2 – 2.5
Iron (Fe)^a^	0.03 – 4	Phyllochinon (K)	0.025
Manganese (Mn)^a^	0.02 – 2		
Chromium (Cr)^a^	0.01 – 0.3		
Zinc (Zn)^a^	0.05 – 2		

*Adapted from [2,3]^a^Heavy metals.

These substances are of nutritional and health importance. Some of the vitamins found in honey include ascorbic acid, pantothenic acid, niacin and riboflavin; along with minerals such as calcium, copper, iron, magnesium, manganese, phosphorus, potassium and zinc. The detailed list of vitamins, minerals, other micronutrients and trace elements found in honey is given in Tables 2 and 3.

**Table 3:** Other chemical elements found in honey*

**Element**	**Amount (mg/100 g)**	**Element**	**Amount (mg/100 g)**
Aluminium (Al)	0.01 – 2.4	Lead (Pb)^a,b^	0.001 – 0.03
Arsenic (As)^a,b^	0.014 – 0.026	Lithium (Li)	0.225 – 1.56
Barium (Ba)	0.01 – 0.08	Molybdenum (Mo)^a^	0 – 0.004
Boron (B)	0.05 – 0.3	Nickel (Ni)^a^	0 – 0.051
Bromine (Br)	0.4 – 1.3	Rubidium (Rb)	0.040 – 3.5
Cadmium (Cd)^a,b^	0 – 0.001	Silicon (Si)	0.05 – 24
Chlorine (Cl)	0.4 – 56	Strontium (Sr)	0.04 – 0.35
Cobalt (Co)^a^	0.1 – 0.35	Sulphur (S)	0.7 – 26
Fluoride (F)	0.4 – 1.34	Vanadium (V)	0 – 0.013
Iodide (I)	10 – 100	Zirconium (Zr)	0.05 – 0.08

*Adapted from [2,3]^a^Heavy metals.

^b^Toxic heavy metals listed amongst the first 20 top hazardous substances in the priority list compiled by ATSDR thus 1 : Ar, 2 : Pb, 7 : Cd; Presence and toxicity in NH can be due to contamination through human error or inimical practices.

The high nutritional profile of honey with wide range of nutrients (although in minute quantities), encourages its use as food. Due to the low quantities of some of the NH’s essential nutrients, it is advisable for adults to take it (NH) in large quantities (70 – 95 g daily) to get the full desirable nutritional and health benefits [19-23].

### Growth

Food is eaten for nourishment, metabolic activities, growth and healthy living. Regular consumption of natural honey gives all these benefits. In fact, honey is a complete meal, as shown in Tables 1 – 3. It contains major components of a meal, and micronutrients that will enhance the digestion and absorption of these major dietary components, as well as those required for metabolism and body functions. We recorded enhanced body weight gain by our rats fed blossom honey in two separate studies at different laboratories in Nigeria and South Africa [4,24]. In 2008, Chepulis and Starkey fed honeydew honey to 8-week old rats for 52 weeks to assess weight gain. These workers show that the growth influence of honey in rodents is partly due to increased bone growth and mineralisation [25], probably due to the calcium content of honey. Our unpublished data from very recent study on NH supplemented rats confirmed this linear growth influence of honey. In his extensive review of the literature, Molan (2001) confirmed the growth stimulating property of honey [18]. He opines from his histological studies on wounds that stimulation of cell growth by honey also enhances NH healing properties.

### Source of antioxidants

The presence of free radicals and reactive oxygen species (ROS) is culpable in the processes of cellular dysfunction, pathogenesis of metabolic and cardiovascular diseases (CVDs) as well as aging. The consumption of foods and substances rich in antioxidant can protect against these pathological changes and consequently prevent the pathogenesis of these and other chronic ailments. Researches indicate that NH contains several important compounds, and these include antioxidants [26,27]. The qualitative and quantitative composition of honey (including the antioxidants constituent and the other phytochemical substances) is a reflection of the floral source as well as the variety of the particular honey. The colour of honey also influences its antioxidant content, as darker honeys are known to have higher amount than lighter honeys [28]. In their analysis of the phytochemical composition of monofloral Cuban honeys, Alvarez-Suarez and co-workers, agreed with this submission and concluded that Cuban honeys contain important phenolic, flavonoid and carotenoid concentrations with high substantial antioxidant capacity [29]. Researchers in California also submitted that human beings can be protected from the damaging effects of free radicals and ROS. The protection is through the absorption of the antioxidants from foods such as honey highly-rich in this important substance called antioxidant. The report of their study in which two buckwheat honey treatments were administered to 37 healthy human adults at the rate of 1.5 g/kg body weight, with corn syrup as control, show increased (p < 0.05) plasma total-phenolic content and plasma antioxidant. Thus, supporting the concept that phenolic antioxidants from processed honey are bioavailable, and they increase antioxidant activity of plasma. They advocated for the substitution of honey in some foods as traditional sweetener for enhanced antioxidant defence system in healthy human adults [27].

### Exercise and athletic performance

The consumption of energy giving substances before, during and after any form of physical exercise improves the individual’s performance and increases the rejuvenation of muscles. This is also associated with dietary supplementation with NH, which provides up to 17 g of carbohydrates for every tablespoon consumed and gives the much needed energy, thus serving as an inexpensive substitute to commercially available sporting activities enhancers. The data obtained from the Sports Nutrition and Exercise Laboratory of one University show that honey can be used effectively instead of glucose for energy replenishment during physical exercise [30]. The physiological actions of NH observed during this performance were a significant increase in heart frequency and a fairly constant blood glucose level. These suggest honey as a better substitute to glucose. Earnest and co-workers improved on this preliminary investigation in another trial by administering low (honey) or high (glucose) glycaemic index (GI) carbohydrate gels on athletes, and testing them on the performance of cyclists travelling a distance of about 65 km [31]. The results of the cycling event show that both the low (honey) and the high (glucose) GI substances caused increase in performance. However, the effect produced by eating NH surpassed that observed in the athletes fed with glucose. This aligns with other previous studies that NH consumption does not compromise metabolic and physical activities [4,25,26]. NH has been shown to decrease blood glucose level in hyperglycaemia such as diabetic subjects [19,20,32-37], plausibly due to the beneficial effects of fructose [32,34-36], and more importantly honey’s several phytochemical constituents [2,37]. A very recent review of the hypoglycaemic effect of honey by some workers conclude that, the synergistic effect of fructose and glucose constituents of honey might contribute to the low glycaemic response after a honey meal [38]. These experimental and clinical trials show that honey is a well-tolerated liquid food.

Furthermore, honey can be an effective carbohydrate source and a better substitute to glucose for exercise and athletic performance, due to its constituent of various classes of sugars. People favour slow-burning sugars for sustenance as energy source during physical exercise. Honey is beneficial in this regard as it releases fructose slowly into the blood stream to produce a sustained energy boost and maintain homeostasis. The other major component of NH apart from fructose is glucose (Table 1). Fructose and glucose are ketose and aldose sugars respectively with chemical structural differences, and consequently different patterns of metabolism, despite both being monosaccharides with quick burning tendency. It is important to note that glucose is rapidly metabolized for absorption into the blood system for energy provision. On the other hand, fructose absorption is slow, and will continue to sustain the individual with energy, while the glucose moiety burns out. The various phytochemical constituents of honey [2] also contribute to the progressive slow rate of fructose metabolism [39]. In addition, honey contains disaccharides such as sucrose and oligosaccharides as well as other trisaccharides (Table 1) that are slow burning sugars. These could facilitate energy replenishment, muscle recuperation and enhancement of performance in athletes nourished with honey, while those relying on glucose for an energy boost might have been exhausted.

### Digestion and absorption

Natural honey contains several enzymes which enhance the digestion of food substances especially carbohydrates such as sugars and starch. The additional benefit of eating honey as a source of energy over the commonly used artificial sugar is that, the major sugar constituents of NH are present as monosaccharides (simple sugars) [2]. Unlike the refined sugar (sucrose) which normally has to undergo the processes of digestion into simpler forms prior to their absorption, these sugar molecules in NH are in pre-digested forms, and can be directly absorbed into the human system. Apart from giving nutrition, the use of honey as a sweetening agent in sweets and desserts is also beneficial. As a sweetener, honey has nutritional advantages over sugar, providing some amount of small nutrients [2,3], which act to aid digestive processes in the body. The gastrointestinal tract (GIT) contains lot of essential and beneficial bacteria, especially Bifidobacteria for the maintenance of life and good health. It has been suggested that one can increase the Bifidobacteria populations in the GIT by consuming foods with rich supply of prebiotics such as natural honey [40,41]. Prebiotics are substances that facilitate the enhanced growth and the biological activity of these good and beneficial bacteria. The consumption of honey is important in human digestion, and this effect is produced by the honey’s constituents of oligosaccharides [40-42]. Several experimental trials involving both *in vitro* and *in vivo* studies have been documented on the importance of dietary supplementation with natural honey on the growth of the beneficial bacteria (bifidobacteria and lactobacilli) and their prebiotic effects in the GIT [40-45]. One comparative study on natural (honey) and artificial (sucrose) sugars shows that honey increased both *in vitro* the beneficial bacteria, lactobacilli, as well as *in vivo* (within the small and the large intestines of experimental rats), while sucrose had no effect [46]. In some cases, the consumption of relatively large amounts of NH (between 70 to about 95 g) can produce a mild laxative effect in people with fructose malabsorption or inadequate absorption [47,48]. Generally, honey has a laxative effect on the digestive system of individuals. Another nutraceutical function of honey is provision of calcium. Honey consumption provides calcium, which is readily absorbable and strengthen bone mass development. This can help reduce the risk of osteoporosis or low bone mass (causative agent of fractures) in old individuals. Research in animal models show that calcium absorption increased correspondingly with increased honey intake [49].

### Children nutrition

There are anecdotal evidences encouraging the feeding of honey to new born babies by some customs and traditions. It is now an established fact that feeding honey to infants will improve memory and growth, reduce anxiety and enhance the children’s performance in later life. In 2009, Chepulis and co workers gave scientific credence to this beneficial practice in their New Zealand behavioural study in animals [50]. They fed 8 weeks old rats with diet supplemented with either honeydew honey or sucrose, and control group with sugar-free diet (all diets patterned after typical New Zealand human diet). These workers noted improved spatial memory and reduced anxiety in the honey-fed rodents better than the other groups over the twelve months trial period. The authors concluded that early introduction of honey diet is beneficial and can improve memory loss and cognitive decline associated with aging [50].

The application of honey in human infant nutrition also revealed some interesting and beneficial observations. The palatability of honey for infants was investigated by Ramenghi and others in 2001, and these workers reported that honey was well tolerated and significantly reduced the crying phases of babies than sterile water [51]. In a review on the importance of honey relative to sucrose in children’s nutrition, honey fed infants were found to have improved haematological profiles and calcium uptake, no digestion problem, lighter and thinner faeces, better skin colour, less susceptibility to diseases, and steady weight gain [52]. These beneficial effects produced by NH when included in infant formula are attributable to its effects in enhancing the gastrointestinal function which include the digestion process. The possible cause is the effect of the carbohydrate constituents, oligosaccharides in NH on intestinal flora of these children [53]. One will be doing well to children by giving honey to replace sweets and other sugary substances they are often inclined to eat.

## Medicinal properties of honey

### Haematology and immunity

Honey has been found to be beneficial to people suffering from anaemia. Ajibola et al. (2007) reported enhanced blood profiles in floral honey-fed adult rats [4]. The study recorded improved haemoglobin concentration (iron constituent of NH played an important role in this), increased erythrocyte count and elevated haematocrit in the honey eaters. In another laboratory, Chepulis (2007) also documented enhanced haematology and immune response in rats fed 10% honeydew honey supplemented diet [54]. The author noted higher lymphocyte count and increased neutrophil phagocytosis in NH-fed rats than control. This aligned with previous research that prebiotics can enhance immune function [55,56] and NH is known to contain the prebiotics, oligosaccharides [4,40,57]. Human subjects administered with two honey treatments in a Californian study show that honey eaters have the benefit of haematoprotection in addition to blood proliferation [27]. The researchers observed that the aqueous portion of the blood (plasma) is protected by honey. This is in agreement with the fact that most of the antioxidant components in processed honey are water soluble. In summarizing the facts that honey can be considered to be a satisfactory immuno-nutrient, some workers opine that the oral administration of natural honey can stimulate and increase the production of antibody during primary and secondary immune responses against the T-cells of the thymus-dependent as well as the thymus independent antigens [58].

### Oral health

The use of NH can promote oral health and wellness. Molan opines that honey with high level of antibacterial activity has the potential to reduce the risk of dental caries [59]. In addition to the carioprotective effect of New Zealand manuka honey (a very potent antimicrobial honey), Molan and co-workers have shown from his extensive work on the influence of honey on oral health that honey prevents dental plaque, gingivitis, periodontics [60]. Other workers in different laboratories have also shown that honey is non-cariogenic or less cariogenic than sucrose [61-64]. The carioprotective effect of honey has been adduced to its antibacterial property, which prevents the growth of the bacteria that can cause dental caries [59,62]. In one electron microscopy study, honey consumption was found to be safer and less inimical to oral health than drinking fruit juice [65]. There was a report of the tooth enamel being eroded just ten minutes after the consumption of fruit juice, while honey ingestion delayed this observation till half an hour after the intake of NH, and this erosion of teeth was not even as prominent or visible as that observed in the fruit juice eaters. The plausible explanation for the less cariogenic effect of honey is the protective role of NH constituents which include calcium, fluoride, phosphorous and other colloidal honey components. In summary, it can be concluded that honey has constituents with cario-protective effect.

### Gastroenterology

Anecdotal evidences advocate the medicinal use of NH as therapeutic agent against the ailments of the GIT in the past [6,66-69]. These are presently being supported by the global medicinal use of NH for the prevention, cure and the treatments of some GIT disorders such as ulcers, gastritis and gastroenteritis [70-77]. Honey has been shown to be a gastroprotective agent. Its potency in inhibiting the activity of *Helicobacter pylori,* that causes gastritis and peptic ulcers have been well documented [78-80]. In experimental rats, NH mitigated gastrointestinal assaults caused by alcohol, ammonia, aspirin and indomethacin [70,71,74,75]. Two mechanisms have been proposed to be responsible for this protective action of honey. The first suggests that this effect is due to the antioxidant properties of honey. NH was found to maintain or enhance the level of non-protein sulfhydyl substances (such as glutathione) in gastric tissue subjected to factors inducing ulceration [70-73]. Similar observation was made when Anzer honey pre-treatment was used to prevent N-ethylmaleimide (NEM) -induced liver damage in rats [81]. The findings imply that depletion of glutathione concentration plays an aetiological role in NEM-induced liver injury, and that the hepatoprotective effect of Anzer honey may be mediated through the sulfhydryl sensitive processes. The authors concluded that honey possess antioxidant properties.

According to some authors, the second mechanism of action being proposed shows that honey intake stimulates the sensory nerves in the stomach, and this proprioceptive effect is in response to capsaicin [70,73]. This mechanism involves the reduction of ulcer index, vascular permeability, and muscular activity of the stomach [77]. Other authors also explained this phenomenon by reporting the mitigating effect of dandelion honey intake against gastric juice acidity by more than 50% [82]. One study reported a slower passage rate of gastric content of saccharides after the intake of NH than that after ingestion of a mixture simulating honey that is glucose and fructose mixture [83], and thus, mitigating diarrhoea. The clinical uses of honey in infants and children revealed shorter duration of diarrhoea caused by bacteria. In the same vein, NH also reduced the pathogenesis and duration of viral diarrhoea unlike that associated with the use of conventional antibacterial therapy [84]. Honey was also found to be beneficial in maintaining blood-sugar levels. In honey, there is little water available to promote the growth of bacteria and yeast. In addition, honey's natural acidity inhibits some pathogens, as it has an amount of hydrogen peroxide and other substances contributing to its antibacterial effect [18].

### Ophthalmology

The use of honey in the treatment of eye diseases is well documented. The ancient people used honey from Attica [85], and Indian lotus honey [86] as curative substances for eye disorders. The Indian locals still use NH as eye drops to cure eye disease till today [87-89]. The Malian people used NH as a tradomedicinal therapeutic agent against measles. The NH was usually applied on the eyes to prevent the scarring effect of the cornea which occurs as a complication of the measles infection [90]. There was an astounding success reported from NH application in clinical trials of 102 patients with different ophthalmological disorders such as (blepharitis, conjunctivitis and keratitis), hitherto not responding to conventional treatment [91]. After the NH application under the lower eyelid like an eye ointment, improvement was seen in 85% of the cases, with no deterioration seen in any of the other 15%. However, a transient stinging sensation and redness of the eye was observed soon after putting honey in the eye, but not enough to halt the treatment in all the 102 cases in the trial. The use of NH application in ophthalmology in Asia and Eastern Europe has also been reported by Molan in the review on NH use in ophthalmology [16]. In one Indian Medical College, an Ophthalmic Surgeon successfully treated bacterial caused corneal ulcers with the topical application of NH [92]. The same paper documented Meier referring to NH being used for the treatment of eyes discharging pus [92]. Molan (1999) also cited Russian authors, Mozherenkov and Prokof'eva that document a review on the use of honey in ophthalmology [16]. The Russian authors observed antibacterial, antifungal and anti-inflammatory actions with the honey application to the eye under the lower eyelid [93]. It has been used for the treatment of burns to the eye caused by chemical and thermal agents, as well as conjunctivitis, and corneal infections. This is usually done by the topical application of the undiluted NH on the affected eyes, or alternatively as a solution containing about 50% water, without any loss of potency [16].

### Metabolic and cardiovascular effects

It has been shown that honey intake ameliorates risk factors of metabolic and cardiovascular diseases in patients and healthy individuals at risk. Unlike refined sugars, diabetic patients can safely and harmlessly eat this natural and sweetest sugar (fructose)-containing product, natural honey. According to Costa-Neto (1999) reported by Santos and Antonini, the Pankararé tribe of Brazil even recommended honey’s use for the treatment of diabetes mellitus, bronchitis, mycosis, and throat aches amidst other ailments [94,95]. Recently in our laboratory, we fed male and female rats with NH or sugar (golden syrup, GS) supplemented diet for 12 weeks from 7 days of age to compare their metabolic response, and see if NH is protective against metabolic syndrome (MetS). This MetS is a condition characterized by abdominal obesity, hyperglycaemia, hypertension and dyslipidaemia, and thus increased susceptibility to diabetes, kidney and heart diseases [96,97]. In male rats, GS significantly increased (p < 0.05) blood levels of metabolic substrates (glucose and triglycerides, TGs); and caused enhanced (p < 0.001) visceral adiposity, hypercholesterolemia, hyperinsulinemia, hepatomegaly and fatty liver. These CVDs and metabolic diseases’ risk factors were not observed in the NH-fed rats in this trial. We concluded that honey is cardio-protective, and its (NH) consumption could not induce MetS [unpublished results]. These results confirmed the conclusion drawn from earlier study that substituting honey for refined carbohydrates was beneficial [40]. Earlier researches from other laboratories and clinical trials further affirmed the metabolic and cardiovascular health significance of eating honey by recording some health profiles. These were reduction in the plasma levels of risk factors which include total cholesterol [19,20]; low density lipoprotein (LDL)-cholesterol [19,20,98]; TGs [19,24,31]; glucose in normal and diabetic patients [19,20]; C-reactive protein [19,20]; while the health indices elevated in the blood were high density lipoprotein (HDL) cholesterol [19,20,25,40]. In addition, other workers recorded higher plasma antioxidants levels in rats nurtured with natural honey relative to fructose-fed rats, and consequently low susceptibility of these subjects to CVDs [40].

### Chemotherapy and wounds management

Honey has antiseptic properties, good for treating burns, infected surgical wounds and ulcers. Glenys Round, a Cancer Specialist and Julie Betts in Waikato Hospital, New Zealand reported excellent results of the therapy from patients with fungating wounds, recalcitrant leg ulcers and pressure sores using the Unique Manuka Factor (UMF)-containing honey known as manuka honey [99]. According to the Specialist, the application of honey dressings was used in these patients (including those with cancer broken through the skin), after failure of healing from conventional treatments which include radiation therapy. Another Researcher successfully treated experimental surgical wounds in Nigerian Dwarf goats with blossom honey. He observed epithelialisation and significantly higher contraction of the NH treated wounds relative to untreated wounds [13]. Other several workers had also used honey for the same purpose in human surgical wards and on experimental animals [100-105]. A comprehensive review of effect of honey on wounds is available elsewhere [16].

### Antimicrobial activity

Natural honey is a very potent broad spectrum antibiotic which most multi-resistant bacteria are found sensitive to (Table 4). Alvarez-Suarez and co-workers confirm this in their report and opine that antimicrobial activity is present in all types of honey [106]. The workers suggest that hydrogen peroxide formation may play an important role as antibacterial natural products for minimizing the invasive effects of bacterial infections in the native monofloral Cuban honeys analysed. Although, the Cuban honeys varied in their chemical constituents’ values, all still exhibit antimicrobial activity against both Gram-positive and Gram-negative bacteria which include *Bacillus subtilis, Pseudomonas aeruginosa, Staphylococcus aureus* and *Escherichia coli*[106]. The bacteria and other micro-organisms infections responsive to NH treatment are listed in Table 4.

**Table 4:** List of Bacteria and other Organisms found to be sensitive to honey*

**Actinomyces pyogenes**	**Pseudomonas aeruginosa**
Bacillus anthracis	Rubella virus
Campylobacter coli	Salmonella cholerae-suis
Campylobacter jejuni	Salmonella typhi
Candida albicans	Salmonella typhimurium
Corynebacterium diphtheria	Serrata marcescens
Echinococcus parasite	Shigella species
Enterococcus avium	Staphylococcus aureus
Enterococcus faecalis	Streptococcus agalactiae
Enterococcus faecium	Streptococcus dysgalactiae
Enterococcus raffinosus	Streptococcus faecalis uberis
Epidermophyton floccosum	Streptococcus mutans
Escherichia coli	Streptococcus pneumonia
Haemophilus influenza	Streptococcus pyogenes
Helicobacter pylori	Streptococcus uberis
Klebsiella pneumonia	Serrata marcescens
Leishmania parasite	Shigella species
Microsporum canis	Trichophyton mentagrophytes
Microsporum gypseum	Trichophyton mentagrophytes var.
Mycobacterium tuberculosis	Trichophyton tonsurans
Nocardia asteroids	Trichophyton rubrum
Proteus species	Vibrio choleriae

*Adapted from [18,107,108].

In their various studies with about 200 New Zealand (Manuka and non-Manuka) honeys, Molan and co-workers are of the opinion that differences exist in the antibacterial and antifungal activities, although all exhibit potency [107-110]. Other pathogenic microbes reported to be susceptible to honey treatment are *Candida albicansRubella* virus, *Trichophyton mentagrophytes,* and *Leishmania* parasites [109,111-113], as shown in Table 4.

### Adverse effects

NH like any other natural foods can also be exposed to contamination by antibiotics, pesticides, heavy metals and other toxic compounds [114]. These poisonous substances can result from disease control, accidental exposure, environmental hazards and inimical human practices [115,116]. It was reported that European health authorities found lead (Pb) in honey bought from India in early 2010 [115]. The presence of contaminants in Indian honeys was confirmed a year later in a test by the Indian Export Inspection Council. The findings showed the presence of lead and at least two antibiotics in almost 23% of the 362 test samples of honey meant for export [115]. The antibiotics could have been the residual effect of treatment and control of infection in honey-bees. A link to this hypothesis is the almost simultaneous, coincidental control of the bacterial epidemic of foulbrood disease in bee hives by Chinese beekeepers, with the use of several antibiotics manufactured in India for animal use, including chloramphenicol [115]. Medical researchers had shown that treatment of children with chloramphenicol as an antibiotic can cause susceptibility to DNA damage and carcinogenicity. The amount of chloramphenicol found in NH although minute, according public health experts, could cause a severe, fatal reaction such as aplastic anaemia in about one out of 30,000 people [115]. This led to the ban placed on honeys originating from China by the United States of America agency, Food and Drug Administration (FDA).

The high concentration of heavy metals in honey can be a source of illness to human beings. Heavy metals are chemical elements with a specific gravity that is at least five times the specific gravity of water. The heavy metals that are of concern in NH production and apicultural practices are listed in Tables 2 and 3. In small quantities, some heavy metals are nutritionally essential for a healthy life. These are referred to as the trace elements, and are listed under minerals in Table 2. The non-essential elements associated with NH are often absent or present in very minute and insignificant levels. These heavy metals of no biological and chemical significance identified in Table 3 are cobalt, molybdenum and nickel. However, there are certain heavy metals present in honey above permissible levels by pollution standards leading to toxicity [116]. The poisoning is due to these heavy metals’ inability to be metabolized by the body, thus leading to their being accumulated to toxic levels within the human or animal soft tissues without being degraded or destroyed. It has been reported that Pb can cause damage of brain, kidney, nervous system and red blood cells. The other health problems caused by heavy metals toxicity include headache, metabolic abnormalities, respiratory disorders, nausea and vomiting [117].

The use of agrochemicals in growing of flowers causes contamination of nectar with heavy metals [2], such as arsenic (As), cadmium (Cd) and Pb. These three heavy metals (As, Cd, Pb) have been identified in the priority list of top 20 hazardous substances compiled by an agency of the United States of America Department of Health and Human Services, known as The Agency for Toxic Substances and Disease Registry (ATSDR) in 2001. [118]. According to ATSDR, As is the most hazardous and toxic substance, being the first on the Agency’s priority list, closely followed by Pb as second, while Cd ranked seventh on the list. Arsenic poisoning could result from environmental contamination through the use of pesticides and paints manufacturing [118]. The limited sources of As pollution might be the reason for lack of As contaminated honey cases being reported, despite the severity of its hazard. Cd contaminated honey could be as a result of its (Cd) use in Pb and zinc (Zn) mining; use of pesticides; and improper handling and disposal of old, used Cd batteries. Nonetheless, Cd concentration in NH is 0 to 0.0001 mg/100 g honey (Table 3), probably due to its similar limited sources of environmental pollution like arsenic, and legislation on protection against occupational health [118]. These showed that this natural food (NH) can be said to be safe from two of the three most hazardous contaminants. However, it is known that heavy metal poisoning results from long term low level exposure to contaminants [114,118]. Hence, no effort should be spared at ensuring the availability of wholesome NH devoid of toxic heavy metals especially Pb, which is the most commonly encountered top hazardous substance of the three identified by ATSDR as being associated with NH.

The other factors causing metal contamination of NH are the methods of honey harvesting, processing and storage. Most small scale local beekeepers use low cost metallic containers due to low purchasing power [115]. Thus, the acidic nature (pH 3.2 – 4.5) of NH (Table 1) corrodes the metal containers [115,119]. Lead is the most documented of all heavy metals causing honey contamination [114-116], due to the various health problems it causes. The possible reason for its presence in different types of honey from several geographic locations could be as a result of the high concentration of Pb in the air due to oil extraction and automobile exhaust emissions [116]. Another environmental factor causing high Pb presence in honey could be due to poor waste disposal of paints, printing materials such as ink, as well as used dry batteries in some places [114]. The other heavy metals of public health importance found in NH apart from As, Cd and Pb are chromium (Cr) and zinc (Zn) (Table 3). The contact of honey with stainless steel surfaces during honey production can also generate a high Cr content, due to the corrosive effect of honey acidity. It has also been documented that NH storage in galvanized containers can be a source of Zn contamination [114,120]. Therefore it is important to take into account the type and quality of equipment used to produce and store honey after harvesting as the possible sources of honey contamination with heavy metals. In addition, the increasing overwhelming demand of this natural product necessitates the promotion of all feasible activities towards ensuring quality [115]. This would increase the production of residue-free and wholesome honey for domestic and international consumption [121]. The safety of consumers and the high global demand for quality honey make international legislation on the food imperative.

The concern of Paediatricians, microbiologists, nurses, Care-givers in health centres and maternity homes as well as other health professionals for infants is in regard to the presence of toxic bacteria, *Clostridium* (*Cl.*) *botulinum* in natural honey. Since this bacterium may be present in natural foods, and honey mostly being a non-sterilized packaged food, the risk of contamination cannot be ruled out. In order to avoid the exposure of infants with immature GIT to the risk of contamination with *Cl. botulinum* and its toxin-producing spores, it is advisable not to feed infants that are below one year old with raw honey. However, in order to alleviate the concern of introducing Clostridial infection through this natural product, honey can be sterilized by gamma irradiation without any loss of NH’s properties [60]; thus, preventing contamination without reducing its nutraceutical value and potency.

## Honey and wealth

### NH Production and consumption

Natural honey production is of high economic importance globally. The cost of honey production is minimal compared with the high returns on the investment. It has been estimated that the global market for wound care annually is between two to six billion US dollars. If part of this huge sum is used to procure the recently developed hi-tech honey dressings, it will go a long way to improve honey production. However, little attention is given to this high-yielding enterprise known as apicultural practice. According to the information on honey production and consumption available at http://www.apiservices.com/, the present annual world honey production is about 1.2 million tons, which is less than 1% of the total sugar production. The consumption of honey differs strongly from country to country. The major honey exporting countries China and Argentina have small annual consumption rates of 0.1 to 0.2 kg *per capita*. NH consumption is higher in developed countries, where the home production does not always meet the market demand. In the European Union, which is both a major honey producer and importer, the annual consumption *per capita* varies from medium (0.3 – 0.4 kg) in Denmark, France, Great Britain, Italy and Portugal to high consumers (1.0 – 1.8 kg) in Austria, Germany, Greece, Hungary and Switzerland. In countries such as Australia, Canada and United States of America, the average *per capita* consumption is 0.6 to 0.8 kg/year [122]. According to FAO (Food and Agriculture Organization of the United Nations) reports of 2005, China is the world largest producer of honey. Other top producers of natural honey are Argentina, Turkey and the United States of America [123]. The significant regional producers of honey include Turkey (ranked third worldwide) and Ukraine (ranked fifth worldwide) [7,124]. Mexico is also known as an important NH producer, providing about 10% of the world's supply. There is urgent need to increase NH production by all countries worldwide to meet the high global demand of honey, which is adjudged an important substitute of refined sugars and conventional medicaments.

### Apicultural practices

Apiculture is the practice of keeping honey bees (*Apis indica, Apis mellifera*) for its products such as NH, wax, propolis, pollens, cerume (a mixture of wax and propolis) etc. It has been shown that NH and its by-products are natural resources, yet to be taken full advantage of as economic earner of little investment with huge returns [121-125]. However, apart from honey and its by-products used for nourishment and medicine, the honey-bees and larvae also serve as source of protein for some people [94,95,125]. These communities harbour stingless bees, a South American species restricted to Brazil, Paraguay, and Columbia [95]. According to Bertoni reported by [95] this species are the best honey producer, but the honey is always somewhat acidic [126], good as an antibiotic. Ethno-entomologists should exploit the trait of honey prolificacy of this species, and also control the acidity of their product (for use as food). This will afford production of more honey, devoid of undesirable quality. Apicultural practices can improve economic earnings of most countries especially in the sub-Sahara African countries and other parts of the globe with forest reserves. Previous studies have shown that bees are integral inseparable part of the ecosystem. According to Costa-Neto 1998, 1999, there are no forests without bees, and there are no bees isolated from the forests [94,126]. Bee keeping can improve small scale and cottage industries, boost foreign earnings from export especially by sub Saharan African countries with vast expanses of unused land spaces and forest reserves.

## Conclusion

The intake of honey as food and medicine resulted in high nutritional benefit and therapeutic promise. The botanical origin plays prominent roles on the bioavailability of NH phytochemical compounds, which consequently has effects on the biological activity of honey. However, irrespective of the floral source, variety and number (mono, polyfloral or blended); honey type (blossom or honeydew honey, Manuka or non-Manuka); concentration (diluted or undiluted); bee (sting or stingless), all contain antioxidants and exhibit various degree of biochemical activities attributable to NH’s potency and value as a nutraceutical agent. The sources and adverse effects of NH contamination identified should be prevented. This could promote the availability of residue-free honey and a wholesome natural product for domestic consumption and international market. This would also help to prevent health problems associated with NH toxicity, especially the most commonly encountered Pb poisoning. Apicultural practices should be encouraged and beekeeping increased especially in countries with avalanche of forests. This will increase NH production worldwide and boost the availability of honey, which is adjudged an important substitute of refined sugars and conventional medicaments. Furthermore, this will facilitate NH’s use as a cheap source of energy nutrient, and as an alternative and economical medicament for most ailments. The stingless bees hitherto restricted to Brazil could be introduced to other parts of the world. The Ethno-entomologists, other Scientists and Apiculturists have roles to play in this development. This will improve apicultural practices especially among people who are sceptical of harm from sting honey bees. The cost of honey production will reduce, and domestic consumption as well as income from export will increase.

## Abbreviations

As, Arsenic; ATSDR, Agency for Toxic Substances and Disease Registry; Cd, Cadmium; Cr, Chromium; CVDs, Cardiovascular diseases; GI, Glycaemic index; GIT, Gastrointestinal tract; GS, Golden syrup; HDL, High density lipoprotein; LDL, Low density lipoprotein; MetS, Metabolic syndrome; NEM, N-ethylmaleimide; NH, Natural honey; Pb, Lead; ROS, Reactive oxygen species; TGs, Triglycerides; UMF, Unique Manuka Factor; Zn, Zinc.

## Competing interests

The authors declare that they have no competing interests.

## Authors’ contributions

KHE mooted the idea of the review; AA carried out the literature search; and AA, KHE and JPC wrote the paper. All authors read and approved the final manuscript.

## Authors’ information

AA, Senior Lecturer, Department of Physiology, Faculty of Basic Medical Sciences, Olabisi Onabanjo University, Ikenne campus, Ikenne 121002, Ogun State, Nigeria. KHE, Associate Professor, School of Physiology, Faculty of Health Science, University of the Witwatersrand, 7 York Road, Parktown 2193, South Africa. JPC, Senior Lecturer, Department of Veterinary Anatomy and Physiology, Faculty of Veterinary Science, University of Pretoria, Onderstepoort, South Africa.
